# Usability and Feasibility of PIERS on the Move: An mHealth App for Pre-Eclampsia Triage

**DOI:** 10.2196/mhealth.3942

**Published:** 2015-04-17

**Authors:** Joanne Lim, Garth Cloete, Dustin T Dunsmuir, Beth A Payne, Cornie Scheffer, Peter von Dadelszen, Guy A Dumont, J Mark Ansermino

**Affiliations:** ^1^Department of Anesthesiology, Pharmacology and TherapeuticsThe University of British ColumbiaVancouver, BCCanada; ^2^Department of Mechanical and Mechatronic EngineeringStellenbosch UniversityStellenboschSouth Africa; ^3^Department of Obstetrics and GynaecologyThe University of British ColumbiaVancouver, BCCanada; ^4^Department of Electrical and Computer EngineeringThe University of British ColumbiaVancouver, BCCanada

**Keywords:** pulse oximetry, mHealth app, predictive model, usability analysis, design methodology

## Abstract

**Background:**

Pre-eclampsia is one of the leading causes of maternal death and morbidity in low-resource countries due to delays in case identification and a shortage of health workers trained to manage the disorder. Pre-eclampsia Integrated Estimate of RiSk (PIERS) on the Move (PotM) is a low cost, easy-to-use, mobile health (mHealth) platform that has been created to aid health workers in making decisions around the management of hypertensive pregnant women. PotM combines two previously successful innovations into a mHealth app: the miniPIERS risk assessment model and the Phone Oximeter.

**Objective:**

The aim of this study was to assess the usability of PotM (with mid-level health workers) for iteratively refining the system.

**Methods:**

Development of the PotM user interface involved usability testing with target end-users in South Africa. Users were asked to complete clinical scenario tasks, speaking aloud to give feedback on the interface and then complete a questionnaire. The tool was then evaluated in a pilot clinical evaluation in Tygerberg Hospital, Cape Town.

**Results:**

After ethical approval and informed consent, 37 nurses and midwives evaluated the tool. During Study 1, major issues in the functionality of the touch-screen keyboard and date scroll wheels were identified (total errors n=212); during Study 2 major improvements in navigation of the app were suggested (total errors n=144). Overall, users felt the app was usable using the Computer Systems Usability Questionnaire; median (range) values for Study 1 = 2 (1-6) and Study 2 = 1 (1-7). To demonstrate feasibility, PotM was used by one research nurse for the pilot clinical study. In total, more than 500 evaluations were performed on more than 200 patients. The median (interquartile range) time to complete an evaluation was 4 min 55 sec (3 min 25 sec to 6 min 56 sec).

**Conclusions:**

By including target end-users in the design and evaluation of PotM, we have developed an app that can be easily integrated into health care settings in low- and middle-income countries. Usability problems were often related to mobile phone features (eg, scroll wheels, touch screen use). Larger scale evaluation of the clinical impact of this tool is underway.

## Introduction

### Background

Pre-eclampsia is generally defined as the onset of proteinuric gestational hypertension [1,2]. Pre-eclampsia remains a leading cause of maternal morbidity and mortality, particularly in low- and middle-income countries (LMICs), in which more than 99% of pre-eclampsia related maternal deaths occur [3]. In LMIC settings, the difficulty in managing women with pre-eclampsia is made greater by delays in identification of women with pre-eclampsia and a lack of adequately skilled maternity care providers [4]. As such, clinical tools for the identification and monitoring of these pregnancies are required [1,5].

PIERS on the Move (PotM) [6,7] is a low-cost, easy-to-use, mobile health (mHealth) app that has been developed to aid frontline health workers in making decisions around management of women with pre-eclampsia. PotM is based on a decision model that combines accurate risk prediction of maternal adverse outcomes associated with pre-eclampsia (miniPIERS [Pre-eclampsia Integrated Estimate of RiSk]) with World Health Organization (WHO) recommendations for the management of pre-eclampsia [8].

The PotM app guides the health worker through a standardized process of antenatal assessment, including measurement of blood pressure, dipstick proteinuria and symptoms. The standardization of care resulting from this guided process and addition of blood pressure measurement as routine practice, which is critical to diagnosis of hypertensive disorders in pregnancy, represents a significant potential to improve clinical care in low-resourced settings. In addition, the PotM app allows integrated measurement of oxygen saturation (SpO_2_), a vital sign previously demonstrated to have significant association with risk of adverse maternal health outcomes in women with pre-eclampsia [9]. The PotM app uses an integrated pulse oximetry sensor (Phone Oximeter) to measure SpO_2_ [10]. The interface and data collection techniques of PotM were designed and developed for use by trained frontline health workers, nurses, and midwives in Africa and South Asia where the burden of pre-eclampsia is greatest. To ensure a user centric design and development process we undertook two usability studies in South Africa; one at Tygerberg Hospital, Cape Town and the second at Frere Maternity Hospital, East London.

Previously, we have described the motivation, design, and technical development of two versions of the PotM mobile app for the diagnosis and management of pregnant women with pre-eclampsia [7]. The purpose of this manuscript is to describe the feasibility and usability evaluation process of the development of the first version of these apps. The usability studies allowed for iterative improvement of the app into a version that is simple, intuitive, and easy-to-use.

### The miniPIERS Model

The miniPIERS model [6] was developed to reduce adverse pregnancy outcomes by providing community-based health workers in low-resourced settings with an evidence-based and low-cost tool to improve diagnosis and initial management of pre-eclampsia. The model uses the demographics (gestational age at presentation), clinical signs (blood pressure and dipstick proteinuria), and symptoms (chest pain or dyspnoea, headache or visual disturbances, vaginal bleeding with abdominal pain) that are more readily available in low resourced settings. Using a threshold of miniPIERS-calculated predicted probability of ≥25% defines a high-risk population with 85.5% accuracy [6]. The risk of developing a complication of pre-eclampsia is more than 30 times greater when the SpO_2_ is <93% [11].

### The Phone Oximeter

The other component integrated into the PotM system is the Phone Oximeter [10], which consists of a mobile phone app that guides users to measure accurate instantaneous oxygen saturation from a connected pulse oximeter. The Phone Oximeter has been optimized for use in the PotM system as an easy and efficient method for health care providers to perform one-minute spot-checks of SpO_2_ [12].

### mHealth Tools

The PotM app uses the massive health care delivery opportunity offered by mobile devices and networks in the developing world. Initial evaluations of mHealth programs to improve maternal health in low-resourced settings have been positive with demonstrated improvements in women’s levels of access of care [13]. mHealth helps to overcome access limiting factors, such as distance to services, social marginalization, and the paucity of skilled medical personnel and finances. Mobile devices are ideal for improving health care delivery. Their popularity and near-ubiquity enables delivery of interventions to large numbers of people, while their mobility allows advanced mHealth apps to be available at any time and place. Open Data Kit (ODK) is an example of a popular mhealth information system that is freely available for download. Evaluation of ODK has shown that mHealth tools can reduce health care costs while increasing the quality and efficiency of care [14].

A study into the usability factors of four different mobile devices for accessing health care information showed that the specific device used for the app played a major role in user satisfaction as well as efficiency [15]. The conclusion was that the future of mHealth app design should place particular importance on interface quality. There are other important considerations when designing an mHealth tool, such as data security and data synchronization. These were previously described for PotM by Dunsmuir et al [7].

### Design Considerations for the Usability of PIERS on the Move

#### System Constraints

The aim was to develop and implement an app for use in low-resourced settings where no formal health care system is available and electricity or a reliable cellular data connection is nonexistent. Therefore, the app was required to make onsite treatment recommendations. Synchronization of the data with the central server was to occur when cellular or wireless connectivity was available.

The primary target user group was frontline care providers (nurses, midwives, community health workers) who might provide care for pregnant women in the community in rural health care facilities or in a hospital setting. The level of education of these users can vary significantly and the system was kept as simple as possible while still being comprehensive to ensure usability at all levels of intended use.

#### Usability Testing Objectives

The usability test objectives were to: (1) exercise the app under controlled test conditions with representative users; (2) establish baseline user performance and user-satisfaction levels of the user interface; (3) determine design inconsistencies and usability problem areas within the user interface and content areas.

Potential sources of error may include: (1) navigation errors–failure to locate functions, excessive keystrokes to complete a function, failure to follow recommended screen flow; (2) presentation errors–failure to locate and properly act upon desired information in screens, selection errors due to labeling ambiguities; (3) control usage problems–improper entry field usage.

##  Methods

### System Design

The PotM full system consists of a client app running on the user’s mobile phone [7], a REDCap [16] database server running a web data collection app, and a web-interface that allows users or supervisors to enter additional follow-up information on patients. The client app was the focus of the usability studies.

PotM was developed using the *LambdaNative* framework, which allows rapid prototyping of the developed apps to run on either Google Android or Apple iOS operating systems [17].

### Hardware Specifications

For the usability studies, the app was installed on iPod Touch (4^th^ generation model; Apple Inc, Cupertino, California, United States) and iPhone 3GS (A1303 model; Apple Inc) devices. The device was hardwired via a serial connection to the dock connector of a US Food and Drug Administration certified 16-bit OEM NoninXpod (Nonin Medical Inc, Plymouth, Minnesota, United States) pulse oximeter processing module. The Xpod pulse oximeter module provides the photoplethysmograph (PPG) waveform and the processed trend values for the SpO_2_ and heart rate (HR).

### User Interface

PotM supports the ability to input, view, and edit the patient history, clinical information, and current symptoms. The app also allows for the viewing of past evaluations and the monitoring and viewing of prescribed medications. All entries are logged with timestamps to ensure data consistency. The expected date of delivery is calculated from ultrasound measurements, last menstrual period or fundal height, in order of reliability when available.

The user interface enforces one minute measurements of oxygen saturation from the pulse oximeter using a color-coded signal quality indicator and progress bar, repeated measurements of blood pressure (2 measurements if consistent or 3 if inconsistent) and dipstick urine protein measurement [7].

### Usability Evaluation

The evaluation of the basic interface functionality, workflow, and navigation was conducted in a series of participatory design groups that included investigators, research staff, and potential end-users. From these design groups, we created the initial prototypes for formal usability evaluation. Two usability studies were performed with the potential end-users. Each step in the development process used the findings of the previous, thus iteratively improving on the design and features available in the app: usability study 1: evaluation by advanced midwifery students at Tygerberg Hospital (Cape Town, South Africa); usability study 2: evaluation of the next iteration by maternal nursing staff at Frere Maternity Hospital (East London, South Africa).

After institutional ethics approval, subject participants were recruited at each of the sites. All nurses and midwives involved in maternity care at the participating sites were invited to participate and the final selection of participants was based on availability on the day of study. Our general pool of potential subjects was chosen as they would be representative of the future end-users of the app; nurses and midwives. Participation was voluntary and participants provided written informed consent. A minimum of 10 participants were selected for each usability evaluation; the suggested sample size for usability testing [18].

The evaluation of the device was performed in a quiet environment (closed room with no distractions). Participants completed a demographic questionnaire (gender, age range, use of mobile phone/personal computer). A facilitator was seated next to the participant and directed the interaction with the hardware and app. All the sessions were videotaped with the camera positioned such that only the hands of the participant and the devices were visible. The facilitator introduced the list of tasks to be completed. Participants were instructed to delay exploratory behavior outside the task flow until after the specific tasks were completed. Time-on-task measurement began when the participant started each of the specified tasks and at the end of each task; an observer recorded the duration of each task.

The facilitator instructed the participant to think aloud [19] during the task and encouraged expression of thought processes during the task performance. The observer entered the user behavior, user comments, and system actions on paper-based data logging forms.

A post-task questionnaire and interview was used to elaborate on the task session. A computer system usability questionnaire (CSUQ) [20] was completed by the participant at the end of the session. Each question was evaluated on a scale of 1 to 7, where 1 and 7 respectively correspond to “strongly agree” and “strongly disagree”.

### Metrics

Scenario completion success rates, time-to-completion of the tasks, specific user comments, error rates, and subjective evaluations were collected for each task.

Each task emulated a typical step in using the PotM platform and required that the participant obtain or input specific data. The tasks were considered complete when the participant indicated that the task’s specific goal was obtained (whether successfully or unsuccessfully) or the participant requested and received sufficient guidance to score the scenario as either “help required” or “critical error”.

### Usability Tasks and Evaluation

The usability tasks (provided as a paper script) (Textbox 1) were derived from test scenarios developed from the specific functionality of the system as well as with the assistance of subject-matter experts. Due to the range and extent of functionality provided in the app, and the time limitations of the participants, the tasks consisted of the most common and more complex of the available functions.

**Usability tasks performed.**

1. Start the PIERS on the Move app
2. Log into the system
3. Enter contact details
4. Enter patient characteristics
5. Enter clinical history
6. Start a new evaluation
7. Enter symptoms
8. Enter blood pressure
9. Record pulse oximetry and heart rate
10. Enter patient outcomes
11. Record management advice provided
12. Enter medications
13. End evaluation
14. Search for the existing patient
15. Enter new patient data as provided
16. Log out of the system

##  Results

### Early Usability

**Figure 1:**
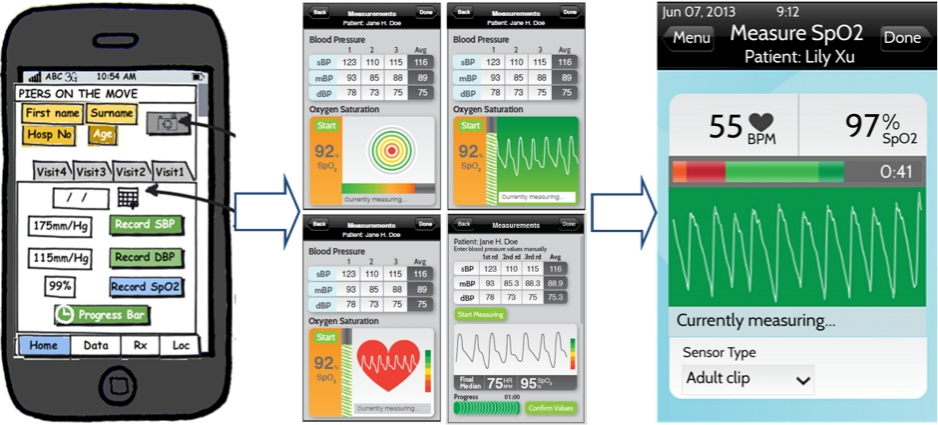
Evolution of the pulse oximetry module.

Participatory sessions involved the generation of a series of wireframe mock-ups using Balsamiq (Balsamiq Solutions, LLC, Sacramento, California, United States) with the basic input variables and forms for each module (Figure 1). Once the mock-ups had been evaluated and optimized, full technical specifications and a final prototype were created (Figure 1). Following additional input from the design team and users, the prototype was implemented in the software framework and tested to ensure it met the required specifications.

The majority of participants were between 31 and 50 years of age (31 of 37 subjects) and were frequent users of cellphones with a minority using mobile phones. There were fewer participants in Study 1 than Study 2 who had never used a mobile phone (n=2 and n=8) or personal computer (n=0 and n=5). All of the participants were female.

### Usability Study 1 – Tygerberg Hospital

**Figure 2:**
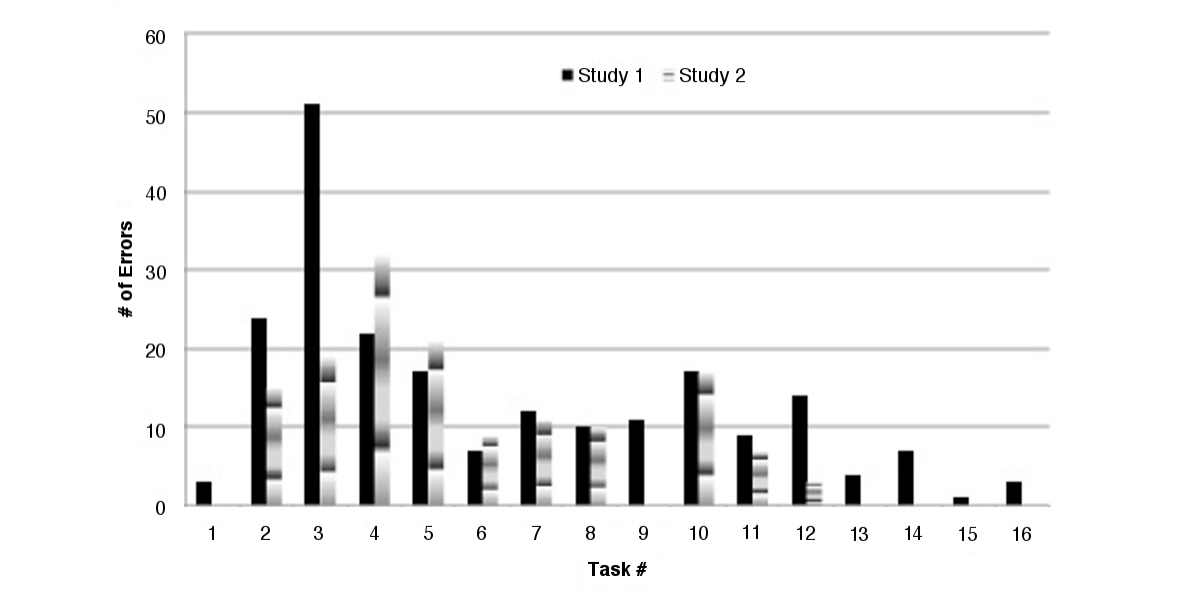
Number of errors obtained during Study 1 & Study 2.

Fifteen volunteers participated in the first study. There were a total of 212 errors in Study 1 (Figure 2).

**Figure 3:**
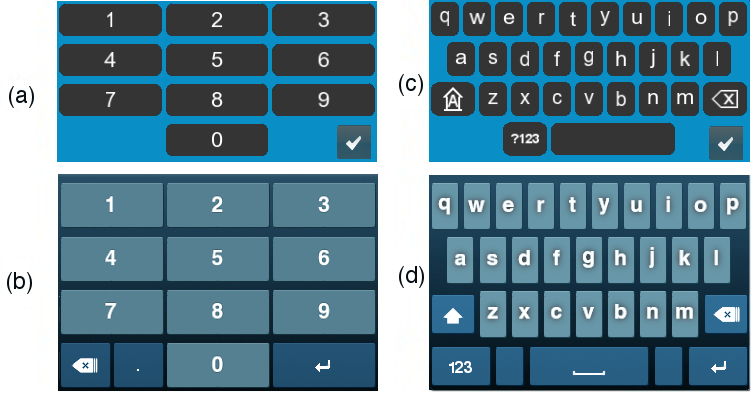
Redesign of the on-screen keypads: (a) & (b) two evolutions of the number pads; (c) & (d) two evolutions of the keypad.

Errors caused by the on-screen keypad and date wheels were the most common complaint. Participant feedback on the use of the keypad were directed to the layout of the keys and included: “keys are too small”, and “wrong button keeps getting pressed”. Whereas feedback concerning the date wheels were typically physics-based and included: “wheels are too sensitive”, and “I don’t understand how to change the numbers on the wheels”. Based on this feedback, both the keypad and date wheels for the app were redesigned. The keypad was enlarged to make all keys easier to press (Figure 3).

In addition, the interaction technique of the date wheels was adjusted to make the date selection easier. Originally, it was only possible to change the value on a date wheel by dragging your finger up or down and the speed of this drag determined how quickly it rotated. The speed of the rotation was faster than the actual drag in order to reach values faster and without multiple dragging of the finger. Once the finger was lifted, the rotation of the wheel stopped. This was found to be confusing and the interaction technique was redesigned. In the redesigned date wheel, the wheel turns at the same speed as the drag but when the finger is lifted, it keeps turning and then gradually slows to a stop. This enables the user to use a quick flicking motion to start the wheel spinning and they can potentially stop it early by touching it again. This more closely matches the physics of a real world spinning wheel.

### Usability Study 2 – Frere Maternity

Evaluations were done by 22 nurses and midwives working at Frere Maternity Hospital (East London, South Africa).

The majority of errors were due to the learning curve required to master the use of a mobile phone or touch screen device for the first time. Typical errors included “why are the keyboard keys in that layout”, or “how do I move up and down on a touch screen”. These errors were quickly corrected once the participant was shown how to perform the function.

Errors were also evaluated in relation to the specific tasks they affected. A summary of the errors for Study 1 and Study 2 can be found in Table 1.

One recurring error was that the date wheel was “too difficult to use” (as seen in the increase in errors compared to Study 1 for Task 4). This occurred frequently even after redesign of the interaction technique of the wheel. This was mainly due to the participants (who are also our target end-users) in rural settings not being familiar with operating smart devices with touch screens and rotating wheel date selectors. The date wheels were subsequently removed, and replaced by up and down arrows to select day, month, and year. The arrow buttons can be held down to enable faster cycling of values, although this cycling is at a set speed. The arrow buttons do not enable as quick date selection as the wheels, but they were easier to understand for new mobile phone users.

**Table 1:** Errors in Study 1 and Study 2: Navigation errors–failure to locate functions, excessive keystrokes to complete a function, failure to follow recommended screen flow; Presentation errors–failure to locate and properly act upon desired information in screens, selection errors due to labeling ambiguities; Control usage problems–improper entry field usage.

Errors		Study 1	Study 2
**Navigation errors**		36	5
	Could not locate or use navigation button	36	5
**Presentation errors**		84	45
	Could not locate data entry feature–missed it	57	33
	Feature did not appear how user expected it to	15	5
	Reason for feature not understood	12	7
**Control usage errors**		92	92
	Had trouble using data entry feature	85	80
	Entered data in wrong location	5	10
	Data entry was not done as user would like it to be	2	2
Program crashed		0	2
Total errors		212	144

### CSUQ Results

The overall satisfaction with the use of the PotM app as measured by the CSUQ was good (lower values are better) and improved in the subsequent study; Study 1 and Study 2 median (range) values are 2 (1–6) and 1 (1–7), respectively. For example, CSUQ statement “I can effectively complete my work using this interface” had a mean of 3.7 in Study 1 and 1.9 in Study 2. “I feel comfortable using this interface” had a mean of 2.7 in Study 1, and 1.6 in Study 2.

### Time on Task

**Figure 4:**
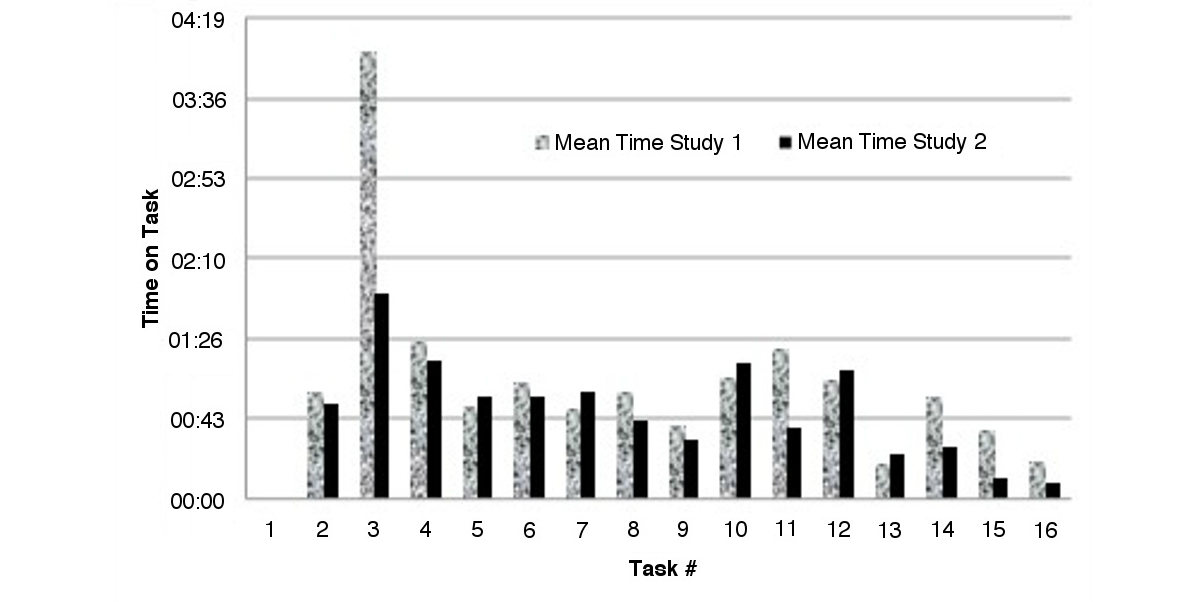
Comparison of Study 1 & 2 time-on-tasks.

There were marked improvements in almost all of the task times with subsequent iterations. Although the average technology proficiencies of the participants in Study 2 were lower than those in Study 1, the participants of Study 2 demonstrated a 25% improvement in scenario completion time (Study 1 had a mean of 37 min 41 sec [SD: 9 min 7 sec], Study 2 had a mean of 30 min 07 sec [SD: 6 min 25 sec]) (Figure 4).

The largest decrease in time taken was seen in *Task 3: Enter Contact Details*, which was the biggest use of a keypad for entering (non-numeric) text. The increase in time taken to complete *Task 7: Enter Symptoms* and *Task 13: End Evaluation* was due to the additional functionality added on the screens at this stage of the study. For Task 7, the Symptoms screen had been redesigned to be more ergonomic and to produce an error message if the form was not fully complete. For Task 13 a detailed summary of why the specific treatment recommendation was given at the end of each evaluation.

### Clinical Trial

The feasibility of using the app clinically was evaluated by considering the time taken for data entry, the amount of missing data, the number of incomplete observations, and subjective feedback from a research nurse who performed all of the observations. The clinical study consisted of collecting patient data using the PotM app using a research nurse at Tygerberg Hospital over the period of November 2012-December 2013. Two hundred two women were recruited and enrolled in the study. The nurse performed multiple evaluations of each patient presenting with pre-eclampsia who met the necessary study inclusion criteria. The nurse was blinded to the recommendations given by the app.

Two secondary objectives of the clinical study were to assess the predictive ability of the miniPIERS model in this new cohort and to test if updating the miniPIERS model to include SpO_2_, a variable that had not been available in the original study, improved predictive ability. Blinding was necessary to ensure clinical care was not affected by the recommendations of the model, which would prevent us from properly meeting these secondary objectives. Results of the miniPIERS model evaluation and updating have been published separately [9].

The average time taken to perform an evaluation (equivalent to Tasks 6-12 in the usability studies) was collected from data logs stored automatically by the PotM platform. In total more than 500 evaluations were performed on more than 200 patients. The median (interquartile range) time for an evaluation was 4min 55 sec (3 min 25 sec to 6 min 56 sec).

##  Discussion

### Principal Findings

We conducted usability tests to optimize the design of an integrated mHealth tool for use by nurses and health care providers to evaluate the risk of developing adverse outcomes of pre-eclampsia. The PotM system integrated the Phone Oximeter and the miniPIERS predictive score and facilitated the collection of patient demographic information, symptoms, and clinical observations with a high degree of completeness in an average of less than 5 minutes in a clinical setting.

Nurses and midwives who participated in our study rated the usability high for the integration of these technologies.

The ease of use of mHealth tools is vitally important to the perception and uptake by the community health workers [21]. It would seem the largest barrier during the testing was the general unfamiliarness of using mobile devices with touch screen technology, and the associated functionality (eg, scroll wheels, on screen keyboards). It should be noted that these typical features in most mobile phones actually seem to be common problems for new users and were the cause of the usability problems. Once these features were explained and/or modified, users were able to complete the functions and tasks much more quickly. In Study 2 and then again for the clinical study, these common data entry features were improved and task times decreased. Even though the user subjects in Study 1 had a greater familiarity with mobile phone use, the CSUQ results demonstrate an overall improvement in usability even with less experienced mobile phone users in Study 2. This may have been caused by the improvements we made to the app between the two studies. Overall, the users we worked with were satisifed with the app and thought it would help their fieldwork. The use of the Phone Oximeter was of particular interest to the midwives as that device could be used in many different clincial settings.

### Limitations

The results should be interpreted with caution as we did not test the device with front line health workers in rural setting who may have very limited education or access to technology. The nurses and midwives in our test population were relatively well-educated. Furthermore, the clinical feasibility study involved a single health care professional using PotM at one health care center, which may limit the generalizability of the findings.

### Future Plans

In the Community Level Interventions for Pre-Eclampsia (CLIP) trial [22], community health care workers (CHWs) are using a version of the PotM app during their regular antenatal and postpartum visits to pregnant women. More than 500 CHWs will use the app to assess over 40,000 pregnant women throughout their pregnancies over the next two years. The CLIP PotM version is simplified for ease of use by those who have less medical training than the users of the original version of PotM. Feedback during the training of these CHWs and during the trial itself has led to further updates to the app. For example, we are displaying the PotM recommendations and record whether the pregnant woman accepted each recommendation. The recommendation summary page contains a short description and image for each appropriate recommendation such as “Transport to the hospital within 4 hours” with a pictogram of an ambulance and hospital. Each pair of image and text was meant to be clicked to go to the page containing the accepted or rejected checkbox (and reasons for rejection). Unfortunately this step was often skipped. Thus we redesigned the summary page to make each recommendation description text appear on a button, making it obvious that it could be clicked and adding a popup to warn users if they try to advance without accepting/rejecting each recommendation. Changes like this are easy to implement and can lead to much more accurate and thorough use of the app.

Our future plans include integrating a full health record for pregnant women during antenatal, intrapartum, and postnatal care including the identification and management of postpartum hemorrhage.

Finally, the Audio Phone Oximeter [23], which consists of a pulse oximeter sensor connected directly to the audio port of a phone, is an innovative solution to pulse oximetry on a mobile device. This eliminates the pulse oximeter processing module, which is expensive hardware that performs the processing of the pulse oximeter signals. Instead, the processing required to extract the HR and SpO_2_ from the PPG waveform will be done directly on the mobile device. Additional sensors such as a semi-automated blood pressure cuff and a temperature sensor, which also connect to the audio port of any mobile device, are being developed.

## Abbreviations

CLIP: Community Level Interventions for Pre-Eclampsia
CSUQ: computer system usability questionnaire
HR: heart rate
LMIC: low and middle income country
mHealth: mobile health
miniPIERS: mini Pre-eclampsia Integrated Estimate of RiSk
PPG: photoplethysmogram
PotM: PIERS on the Move
SD: standard deviation
SpO2: oxygen saturation
WHO: World Health Organization
